# Sex at the interface: the origin and impact of sex differences in the developing human placenta

**DOI:** 10.1186/s13293-022-00459-7

**Published:** 2022-09-16

**Authors:** Amy E. Braun, Olivia R. Mitchel, Tania L. Gonzalez, Tianyanxin Sun, Amy E. Flowers, Margareta D. Pisarska, Virginia D. Winn

**Affiliations:** 1 Department of Obstetrics and Gynecology, Stanford University School of Medicine, 240 Pasteur Dr., Stanford, CA 94305-5317 USA; 2 Department of Obstetrics and Gynecology, Cedars-Sinai Medical Center, 8635 West Third Street, Suite 160, Los Angeles, CA 90048 USA

**Keywords:** Sex, Placenta, Differences, Gene expression, Fetal, Transcriptome, Pregnancy complications

## Abstract

The fetal placenta is a source of hormones and immune factors that play a vital role in maintaining pregnancy and facilitating fetal growth. Cells in this extraembryonic compartment match the chromosomal sex of the embryo itself. Sex differences have been observed in common gestational pathologies, highlighting the importance of maternal immune tolerance to the fetal compartment. Over the past decade, several studies examining placentas from term pregnancies have revealed widespread sex differences in hormone signaling, immune signaling, and metabolic functions. Given the rapid and dynamic development of the human placenta, sex differences that exist at term (37–42 weeks gestation) are unlikely to align precisely with those present at earlier stages when the fetal–maternal interface is being formed and the foundations of a healthy or diseased pregnancy are established. While fetal sex as a variable is often left unreported in studies performing transcriptomic profiling of the first-trimester human placenta, four recent studies have specifically examined fetal sex in early human placental development. In this review, we discuss the findings from these publications and consider the evidence for the genetic, hormonal, and immune mechanisms that are theorized to account for sex differences in early human placenta. We also highlight the cellular and molecular processes that are most likely to be impacted by fetal sex and the evolutionary pressures that may have given rise to these differences. With growing recognition of the fetal origins of health and disease, it is important to shed light on sex differences in early prenatal development, as these observations may unlock insight into the foundations of sex-biased pathologies that emerge later in life.

## Highlights

- Placental sex differences exist from early prenatal development, and may help explain sex differences in pregnancy outcomes.
- Transcriptome profiling of early to mid-gestation placenta reveals that immune signaling is a hub of early prenatal sex differences.
- Differentially expressed genes between male and female placenta fall into the following functional associations: chromatin modification, transcription, splicing, translation, signal transduction, metabolic regulation, cell death and autophagy regulation, ubiquitination, cell adhesion and cell–cell interaction.
- Placental sex differences likely reflect the interaction of cell-intrinsic chromosome complement with extrinsic endocrine signals from the fetal compartment that accompany gonadal differentiation.
- Understanding the mechanisms behind sex differences in placental development and function will provide key insight into molecular targets that can be modulated to improve sex-biased obstetrical complications.

## Background

Sex differences emerge early in human development and are detectable from the embryonic stage to parturition [1, 2]. While sex differences in pregnancy outcomes such as fetal birth weight and infant mortality have been recognized for centuries [3], the biological mechanisms through which fetal sex shapes prenatal development remain to be determined. As the interface with maternal circulation and the central coordinator of fetal growth, the placenta is likely to play a starring role in the production of sex-linked prenatal phenomena.

The framing of sex differences in prenatal development generally centers on male vulnerability. Indeed, male fetuses have been reported to be at elevated risk for early preterm birth, term preeclampsia (PE), placental inflammation, premature rupture of membranes (PPROM) and a variety of other gestational complications [4–9]. However, female fetuses show a higher incidence of preterm PE, intrauterine growth restriction (IUGR) and being small for gestational age (SGA) across multiple populations [10–14], suggesting that sex-biased prenatal vulnerability can manifest in unique and context-specific ways [15–20].

### How does sex manifest in the human placenta?

The placenta is a critical determinant of both fetal and maternal health throughout gestation. In addition to providing the interface for the exchange of nutrients and waste, the placenta is also a source of hormones and immune factors that facilitate pregnancy maintenance and fetal growth [21]. During the process of human placentation, trophoblast cells from the outer trophectoderm layer of the blastocyst invade maternal decidua to form the placenta and chorionic membranes. The resulting extraembryonic compartment shares the biological sex of the developing embryo. Because fetal cells can express paternal antigens that are immunologically distinct from maternal cells, the successful establishment of maternal immune tolerance to the fetal “graft” is an essential requirement for successful placentation and pregnancy. If these finely tuned interactions become dysregulated, placental dysfunction can result, leading to complications such as spontaneous abortion, preterm birth, preeclampsia and intrauterine growth restriction [22]. Given the sex differences observed in common gestational pathologies, the sex of the trophoblast and the other cells that compose the placenta is likely to influence the interactions between fetal and maternal cells.

The primary known factor giving rise to sex differences in early embryogenesis is sex chromosome-linked gene expression (23). In addition to differential expression of X and Y transcripts themselves, differences in autosomal gene expression in early embryonic and extraembryonic tissues have been observed and are likely to play a role in sex-biased fetal outcomes. Towards the end of the first trimester, differential expression between the sexes is likely to reflect the interaction of cell-intrinsic chromosome complement with extrinsic endocrine signals from the fetal compartment that accompany gonadal differentiation. Both potential mechanisms will be expanded upon later in this review.

### The dynamic placental transcriptome

Placentas from term pregnancies have been frequently examined for transcriptomic differences based on fetal sex over the past decade, and these studies have revealed widespread differences in hormone signaling, immune signaling, and metabolic functions [24–28]. Sood et al. [24] first observed sex differences in both sex chromosome and autosomal gene expression in term placentas by microarray, identifying JAK-STAT-related immune regulation as a central signaling hub. Osei-Kumah et al. [25] also highlighted sex differences in cytokine signaling in placentas from pregnancies complicated by asthma, as well as glucocorticoid hormone signaling. Cvitic et al. [26] performed the first cell-type specific analysis, isolating and culturing different trophoblast and endothelial cell types from male and female placentas and subjecting them to microarray analysis. TNFα and NFкB signaling pathways emerged as a major node of sexually dimorphic gene expression patterns, showing elevation in male placental endothelium. Sex-linked alterations in pro-inflammatory signaling at the mRNA and protein levels are a theme across multiple studies in both healthy and inflamed placenta [24, 25, 29]. In line with this, cultured male trophoblasts from healthy term placentas produce more TNFα and less IL-10 than female trophoblasts in response to lipopolysaccharide [30].

Beyond differences in inflammatory signaling, a meta-analysis of transcriptome data from term placentas by Buckberry et al. [27] observed 142 genes differentially expressed (DE) between male and female placentas, with > 60% being autosomal, including genes related to gene transcription, cell growth, proliferation, and hormone signaling. Higher female expression from the LHB-CGB cluster was detected, which includes genes involved in placental development, maintenance of pregnancy and maternal immune tolerance of the conceptus. Osei-Kumah et al. [25] and Sedlmeier et al. [28] reported that female placentas at term are more responsive to changes to both maternal inflammation and diet, with male placental gene expression appearing less sensitive to environmental perturbations. Given the central roles of hormonal and immune regulation in the production of pregnancy pathologies like preterm birth, these placental differences likely play a major precipitating role in sex-biased pregnancy complications and fetal outcomes if they are also present in the placenta earlier in gestation.

Differences in transcript and protein abundance in term placentas are informative but given the rapid and dynamic development of the human placenta [31], sex differences that exist at term are unlikely to align precisely with those present at earlier stages when the fetal–maternal interface is being formed and the foundations of a healthy or diseased pregnancy are established. Several groups have performed transcriptomic profiling of the first-trimester human placenta (reviewed by Yong and Chan [32], 2020: Table 1), however fetal sex as a variable in these datasets is often left unreported or, if reported, is not directly analyzed. In the recent years, four studies have examined fetal sex in early human placental development (Table 1) [33–36]. In the following sections, we review these findings and consider the evidence for the genetic, hormonal and immune mechanisms that are theorized to account for sex differences in early human placenta and highlight the cellular and molecular processes that are most likely to be impacted by fetal sex.

**Table 1:** Summary of early human placenta transcriptome studies

First author	Year	DOI	Topic	Method	Bulk tissue/cell	Sampling location	Gestational age of sample	Sex reported? (Y/N)	Sex-based analysis? (Y/N)
Tsui	2004	https://doi.org/10.1136/jmg.2003.016881	Identify placental-specific transcripts in maternal blood	Microarray	Bulk tissue	Chorionic villi	9–12 and 38–40 wks	N	N
Mikheev	2008	https://doi.org/10.1177/1933719108322425	Profile placenta across gestation	Microarray	Bulk tissue	Chorionic villi	6–8 and 15–16 wks	N	N
Founds	2008	https://doi.org/10.1016/j.placenta.2008.09.015	Preeclampsia	Microarray	Bulk tissue	Chorionic villi	10–12 wks	N	N
Rull	2010	https://doi.org/10.1016/j.placenta.2012.11.032	Recurrent miscarriage	Microarray	Bulk tissue	Placenta biopsy (chorionic villi + basal plate)	1st trimester	N	N
Sitras	2012	https://doi.org/10.1371/journal.pone.0033294	Placental development across gestation	Microarray	Bulk tissue	Chorionic villi	9–12 wks	N	N
Pantham	2012	https://doi.org/10.1016/j.jri.2012.03.487	Antiphospholipid antibodies	Microarray	Primary explants	Placental tissue explants	8–8.5 wks	N	N
Ghaffari-Tabrizi-Wizsy	2014	https://doi.org/10.1159/000381766	1st trimester trophoblast vs. 3rd trimester endothelial degradome	Microarray	Primary cell cultures	Chorionic villi (1st trimester), chorionic plate (3rd trimester)	7–10 wks	N	N
Roost	2015	https://doi.org/10.1016/j.stemcr.2015.05.002	Development from early to mid- pregnancy	RNA-seq	Bulk tissue	Amnion, chorion, chorionic villi, decidua, umbilical cord	8.2–9.6, 16–18, and 21–22 wks	Y	N
James	2015	https://doi.org/10.1530/REP-14-0646	Characterize trophoblast subpopulations in early pregnancy	Microarray	Sorted cell (flow cytometry)	Chorionic villi	5.6–12.5 wks	N	N
Leslie	2015	https://doi.org/10.1016/j.ajpath.2015.06.020	Preeclampsia	Microarray	Bulk tissue	Chorionic villi	9–14 wks	N	N
Lassance	2015	https://doi.org/10.1016/j.ajog.2015.02.026	Obesity/Insulin	Microarray	Primary cell culture	Primary cytotrophoblast culture	7–12 wks	N	N
Tian	2016	https://doi.org/10.1002/path.4694	Recurrent miscarriage	Microarray	Primary cell culture	Chorionic villi	6–12 wks	N	N
Söber	2016	https://doi.org/10.1038/srep38439	Recurrent miscarriage	RNA-seq	Bulk tissue	Chorionic villi	6–12 wks	Y	N
Lim	2017	https://doi.org/10.1371/journal.pone.0181155	Placental development across gestation (w/ linked methylation data)	RNA-seq	Bulk tissue	Chorionic villi	1st and early 2nd trimester	N	N
Lim	2017	https://doi.org/10.1186/s12864-017-3993-y	Chromosomal abnormalities	Microarray	Bulk tissue	Chorionic villi	11–13 wks	N	N
Weisblum	2017	https://doi.org/10.1128/JVI.01905-16	Zika virus infection	RNA-seq	Organoid cultures	Primary decidual and chorionic villus organoid cultures	Early- and mid-gestation	N	N
Soncin	2018	https://doi.org/10.1242/dev.156273	Profile placenta across gestation	Microarray	Bulk tissue	Chorionic villi	4–39 weeks	N	N
Liu	2018	https://doi.org/10.1038/s41422-018-0066-y	Characterize placental cell subpopulations	RNA-seq	Single cell w/ pre-sorting (magnetic bead)	Chorionic villi (1st trimester), basal plate (2nd trimester)	8 and 24 wks	N	N
Suryawanshi	2018	https://doi.org/10.1126/sciadv.aau4788	Characterize cells from first-trimester maternal–fetal interface	RNA-seq	Single cell and bulk tissue	Chorionic villi and decidua	6–11 wks	Y	N
Vento-Tormo	2018	https://doi.org/10.1038/s41586-018-0698-6	Characterize cells from first-trimester maternal–fetal interface	RNA-seq	Single cell w/ pre-sorting (flow cytometry)	Chorionic villi and decidua	6–14 wks	N	N
Turco	2018	https://doi.org/10.1038/s41586-018-0753-3	Validate source and source-derived organoid	Microarray	Bulk tissue	Chorionic villi	6–8 and 10–12 wks	N	N
Huang	2018	https://doi.org/10.1016/j.ebiom.2018.11.015	Recurrent miscarriage	RNA-seq	Bulk tissue	Chorionic villi	< 20 wks	N	N
Zhao	2018	https://doi.org/10.19723/j.issn.1671-167X.2019.01.026	IVF	Microarray	Bulk tissue	Chorionic villi	7–8 wks	N	N
**Gonzalez**	**2019**	https://doi.org/10.1186/s13293-018-0165-y	**Fetal sex differences**	**RNA-seq**	**Bulk tissue**	**Chorionic villi**	**10.5–13.5 wks**	**Y**	**Y**
Lee	2019	https://doi.org/10.1016/j.fertnstert.2018.11.005	IVF and non-IVF treatment	RNA-seq	Bulk tissue	Chorionic villi	11–13 wks	Y	N
**Sun**	**2020**	https://doi.org/10.1210/clinem/dgaa503	**Sexually dimorphic crosstalk at maternal–fetal interface**	**Single-cell and bulk RNA-seq**	**Single cell and bulk tissue**	**Chorionic villi and decidua**	**10–13 wks**	**Y**	**Y**
**Braun**	**2020**	https://doi.org/10.1007/s43032-020-00355-8	**Sex differences during fetal androgen exposure**	**RNA-seq**	**Bulk tissue**	**Chorionic villi**	**11–16 wks**	**Y**	**Y**
**Flowers**	**2021**	https://doi.org/10.1093/biolre/ioab221	**Sex differences in microRNA expression in first and third** **trimester human placenta**	**Small RNA-seq**	**Bulk tissue**	**Chorionic villi and placenta collected at delivery**	**10.4–14.5 wks and 36.3–41.4 wks**	**Y**	**Y**

Bolded values indicate studies in which sex-based analysis is reported

### The sex chromosomes

Sex chromosomes account for the earliest, most pronounced, and most reproducible sex differences in gene expression (23). Potential sources of variation related to sex chromosome complement include expression from the Y chromosome in XY males and selective expression from the second X chromosome in XX females.

An early model of sex chromosome gene dosage compensation held that male cells contain one X chromosome and one Y chromosome, while female cells contain one active and one compacted and inactive X known as a Barr body [37, 38]. This inactivation of one X theoretically ensures that XX transcription matches the dosage in XY males, leading to the model: 1 active X → female phenotype, 1 active X + Y → male phenotype. This simple model is complicated by the existence of the two human pseudoautosomal regions (PARs 1&2), which are not inactivated in XX cells [39, 40], and later by the discovery of other X transcripts located outside of the X chromosome PARs that escape inactivation in a variable and cell-specific manner [41]. Extraembryonic tissues share the sex karyotype of the fetus, and genes in all these categories are likely to play some role in producing sex differences in human placenta.

### Pseudoautosomal genes in placenta

Pseudoautosomal regions (PARs) are short nucleotide sequences on the ends of sex chromosomes exhibiting homology between the X and Y. They are not inactivated in XX cells and exhibit X and Y variants that can be distinguished via PCR and high-throughput sequencing. PAR1, the larger and better characterized PAR, is located at the ends of Xp/Yp and contains at least 24 genes encoding proteins involved in functions including transcriptional regulation, RNA splicing, signal transduction, and cell adhesion [42]. PAR2, located at the ends of Xq/Yq, is evolutionarily recent and unique to humans, making humans the only species known to have 2 distinct PARs [43]. Interestingly, both PARs are enriched for genes that underlie immune signaling, including *IL3RA*, *IL9R*, *CSF2RA*, and *CD99* (Table 2). PAR1 gene *ASMTL*, PAR1 pseudogene *CD99P1*, and PAR2 gene *VAMP7* were upregulated in the male placenta at mid-gestation [35], likely attributable to increased expression from the Yq allele. In a sex-based reanalysis of Soncin et al. (21, GEO accession number: GSE107824) both *CD99* and *VAMP7* trended towards upregulation in cytotrophoblasts from across gestation in male fetuses compared to females. In a meta-analysis of sex differences in human term placenta, PAR1 genes were shown to be elevated in males [27], suggesting that this may be a persistent bias throughout development. PAR gene dosage is altered in sex chromosome aneuploidies such as XXY (Klinefelter syndrome) and monosomy X (Turner syndrome), and abnormal gene dosage of PAR genes is thought to contribute to the elevated risk of pregnancy complication and spontaneous abortion in aneuploid sex chromosome karyotype pregnancies [44–46].

**Table 2:** Gene symbols and full gene names of all transcripts mentioned

HGNC gene symbol	HGNC gene name
ARHGEF9	Cdc42 guanine nucleotide exchange factor 9 [HGNC:14561]
ARMCX3	Armadillo repeat containing X-linked 3 [HGNC:24065]
ARMCX6	Armadillo repeat containing X-linked 6 [HGNC:26094]
ASMTL	Acetylserotonin O-methyltransferase like [HGNC:751]
BMPR2	Bone morphogenetic protein receptor type 2 [HGNC:1078]
BRCC3	BRCA1/BRCA2-containing complex subunit 3 [HGNC:24185]
BSG	basigin (Ok blood group) [HGNC:1116]
C1QTNF1	C1q and TNF related 1 [HGNC:14324]
CANT1	Calcium activated nucleotidase 1 [HGNC:19721]
CAPN6	Calpain 6 [HGNC:1483]
CCL13	C–C motif chemokine ligand 13 [HGNC:10611]
CCL3	C–C motif chemokine ligand 3 [HGNC:10627]
CCL4	C–C motif chemokine ligand 4 [HGNC:10630]
CCRL2	C–C motif chemokine receptor like 2 [HGNC:1612]
CD99	CD99 molecule (Xg blood group) [HGNC:7082]
CD99P1	CD99 molecule pseudogene 1 [HGNC:7083]
CDC34	cell division cycle 34, ubiqiutin conjugating enzyme [HGNC:1734]
CHM	CHM Rab escort protein [HGNC:1940]
CIRBP	Cold inducible RNA binding protein [HGNC:1982]
COL1A1	Collagen type I alpha 1 chain [HGNC:2197]
COL4A1	Collagen type IV alpha 1 chain [HGNC:2202]
COX6B1	Cytochrome c oxidase subunit 6B1 [HGNC:2280]
CSF1	Colony stimulating factor 1 [HGNC:2432]
CXCL8	C–X–C motif chemokine ligand 8 [HGNC:6025]
CYB561A3	Cytochrome b561 family member A3 [HGNC:23014]
DDX3X	DEAD-box helicase 3 X-linked [HGNC:2745]
DDX3Y	DEAD-box helicase 3 Y-linked [HGNC:2699]
ECH1	Enoyl-CoA hydratase 1 [HGNC:3149]
EGFL6	EGF like domain multiple 6 [HGNC:3235]
EID2	EP300 interacting inhibitor of differentiation 2 [HGNC:28292]
EIF1AX	Eukaryotic translation initiation factor 1A X-linked [HGNC:3250]
EIF1AY	Eukaryotic translation initiation factor 1A Y-linked [HGNC:3252]
EIF2S3	Eukaryotic translation initiation factor 2 subunit gamma [HGNC:3267]
ENG	Endoglin [HGNC:3349]
ERBB2	erb-b2 receptor tyrosine kinase 2 [HGNC:3430]
FAU	FAU ubiquitin like and ribosomal protein S30 fusion [HGNC:3597]
FCGBP	Fc fragment of IgG binding protein [HGNC:13572]
FN1	Fibronectin 1 [HGNC:3778]
GAA	Alpha glucosidase [HGNC:4065]
GLA	Galactosidase alpha [HGNC:4296]
GPR108	G protein-coupled receptor 108 [HGNC:17829]
GPR137	G protein-coupled receptor 137 [HGNC:24300]
HDAC8	Histone deacetylase 8 [HGNC:13315]
HLA-C	Major histocompatibility complex, class I, C [HGNC:4933]
HSPA4	Heat shock protein family A (Hsp70) member 4 [HGNC:5237]
HSPA5	Heat shock protein family A (Hsp70) member 5 [HGNC:5238]
IFNG	Interferon gamma [HGNC:5438]
IL10	Interleukin 10 [HGNC:5962]
IL1B	Interleukin 1 beta [HGNC:5992]
IL1RL2	Interleukin 1 receptor like 2 [HGNC:5999]
IL36RN	Interleukin 36 receptor antagonist [HGNC:15561]
IL6	Interleukin 6 [HGNC:6018]
INSR	Insulin receptor [HGNC:6091]
IQSEC2	IQ motif and Sec7 domain ArfGEF 2 [HGNC:29059]
ITGA5	Integrin subunit alpha 5 [HGNC:6141]
ITGB8	Integrin subunit beta 8 [HGNC:6163]
KDM5C	Lysine demethylase 5C [HGNC:11114]
KDM5D	Lysine demethylase 5D [HGNC:11115]
KDM6A	Lysine demethylase 6A [HGNC:12637]
LAMA1	Laminin subunit alpha 1 [HGNC:6481]
LAMB1	Laminin subunit beta 1 [HGNC:6486]
LGALS13	Galectin 13 [HGNC:15449]
LGALS14	Galectin 14 [HGNC:30054]
LSM7	LSM7 homolog, U6 small nuclear RNA and mRNA degradation associated [HGNC:20470]
MAGEA4	MAGE family member A4 [HGNC:6802]
MAOA	Monoamine oxidase A [HGNC:6833]
MRPL54	Mitochondrial ribosomal protein L54 [HGNC:16685]
MXRA5	Matrix remodeling associated 5 [HGNC:7539]
NDUFA11	NADH:ubiquinone oxidoreductase subunit A11 [HGNC:20371]
NLGN4Y	Neuroligin 4 Y-linked [HGNC:15529]
NUDT10	nudix hydrolase 10 [HGNC:17621]
OFD1	OFD1 centriole and centriolar satellite protein [HGNC:2567]
OGT	O-linked N-acetylglucosamine (GlcNAc) transferase [HGNC:8127]
PCDH11Y	Protocadherin 11 Y-linked [HGNC:15813]
PORCN	Porcupine O-acyltransferase [HGNC:17652]
PSG8	Pregnancy specific beta-1-glycoprotein 8 [HGNC:9525]
PUDP	Pseudouridine 5'-phosphatase [HGNC:16818]
RABAC1	Rab acceptor 1 [HGNC:9794]
RBM41	RNA binding motif protein 41 [HGNC:25617]
RGS1	Regulator of G protein signaling 1 [HGNC:9991]
RPS4X	Ribosomal protein S4 X-linked [HGNC:10424]
RPS4Y1	Ribosomal protein S4 Y-linked 1 [HGNC:10425]
SCYL1	SCY1 like pseudokinase 1 [HGNC:14372]
SLC16A3	Solute carrier family 16 member 3 [HGNC:10924]
SMARCA1	SWI/SNF related, matrix associated, actin dependent regulator of chromatin, subfamily a, member 1 [HGNC:11097]
SMC1A	Structural maintenance of chromosomes 1A [HG0NC:11111]
SMS	Spermine synthase [HGNC:11123]
SPP1	Secreted phosphoprotein 1 [HGNC:11255]
STS	Steroid sulfatase [HGNC:11425]
TBL1Y	Transducin beta like 1 Y-linked [HGNC:18502]
TGFB1	Transforming growth factor beta 1 [HGNC:11766]
THOC2	THO complex 2 [HGNC:19073]
TIMP2	TIMP metallopeptidase inhibitor 2 [HGNC:11821]
TMEM164	Transmembrane protein 164 [HGNC:26217]
TMEM258	Transmembrane protein 258 [HGNC:1164]
TMSB4X	Thymosin beta 4 X-linked [HGNC:11881]
TMSB4Y	Thymosin beta 4 Y-linked [HGNC:11882]
TNC	Tenascin C [HGNC:5318]
TNF	Tumor necrosis factor [HGNC:11892]
TRAPPC2	Trafficking protein particle complex 2 [HGNC:23068]
USP9Y	Ubiquitin specific peptidase 9 Y-linked [HGNC:12633]
UTY	Ubiquitously transcribed tetratricopeptide repeat containing, Y-linked [HGNC:12638]
VAMP7	Vesicle associated membrane protein 7 [HGNC:11486]
VPS51	VPS51 subunit of GARP complex [HGNC:1172]
WNT3A	Wnt family member 3A [HGNC:15983]
YIF1A	Yip1 interacting factor homolog A, membrane trafficking protein [HGNC:16688]
YIPF6	Yip1 domain family member 6 [HGNC:28304]
ZFX	Zinc finger protein X-linked [HGNC:12869]
ZFY	Zinc finger protein Y-linked [HGNC:12870]
ZMAT1	Zinc finger matrin-type 1 [HGNC:29377]
ZRSR2	Zinc finger CCCH-type, RNA binding motif and serine/arginine rich 2 [HGNC:23019]

### Mechanisms of XX protection in placenta

In regions of the X chromosome outside of the PARs, X-chromosome inactivation (XCI) normalizes gene dosage between XY males and XX females. Prior to XCI, which occurs between implantation and tissue differentiation, X-linked genes in XX cells are expressed from both the paternal and maternal alleles. XCI is essential for the development of XX conceptuses past the early embryonic stage [47] and occurs via epigenetic compaction of either the paternal or maternal X chromosome into inactive heterochromatin. In monotremes and marsupials, X inactivation is imprinted, with the paternally derived X inactivated in every cell. In Eutherians, the process of X inactivation has become more nuanced, sometimes parentally imprinted and other times randomized via stochastic expression of the X-linked non-coding RNA *XIST*. Murine extraembryonic tissues retain the selective paternal X inactivation seen in non-Eutherian mammals, however this imprint is lost and reset in the cells of the inner cell mass, where a new round of random inactivation allows for mosaic paternal and maternal X expression (48–50).

In the human placenta, the existence of parentally imprinted X inactivation has been a topic of controversy. Some reports indicate skewed X inactivation [51–53], and others report unbiased expression that suggests a more random process matching that of the inner cell mass [54, 55]. An archived report awaiting peer review by Phung et al. [56] provides evidence for a clonal model of X inactivation in placenta, suggesting that there are regions of paternal X inactivation and maternal X inactivation with an overall skew towards paternal inactivation. Sampling bias including the timing, the exact tissue compartment and other confounding factors may help to resolve the puzzling and contradictory findings in these studies. Two regions of opposite X inactivation pooled together may appear randomly biallelic, and sampling from only one region may lead to inaccurate reports of single-parent X inactivation. In the future, a single-nucleus approach will be essential to fully understanding XCI in the placenta especially given the multinucleated nature of the syncytium [57, 58].

The evolutionary movement away from strict paternal X inactivation in Eutherians highlights the dynamic balance of selection pressures on placental X expression. While inactivation of the paternal X through strict imprinting may minimize contact between surveilling maternal immune cells and possible foreign paternal antigens, the genetic robustness conferred by mosaicism may provide a survival benefit. Skewed but non-imprinted X inactivation in placenta may reflect a process of internal selection, particularly in tissues where development involves cellular competition for growth factors resulting in differential cell survival [41]. If one parent’s copy of the X proves more advantageous to the survival of a given cell type, those clones will prevail in that tissue and X inactivation will appear skewed toward one parent without the necessity of a priori genomic imprinting [59, 60]. This may help to explain observations of skewed, clonal X inactivation in tissues like the placenta. The wide heterogeneity within and between individual placentas is also suggestive of an ongoing evolutionary process, where different strategies are viable in different contexts [56].

**Fig. 1:**
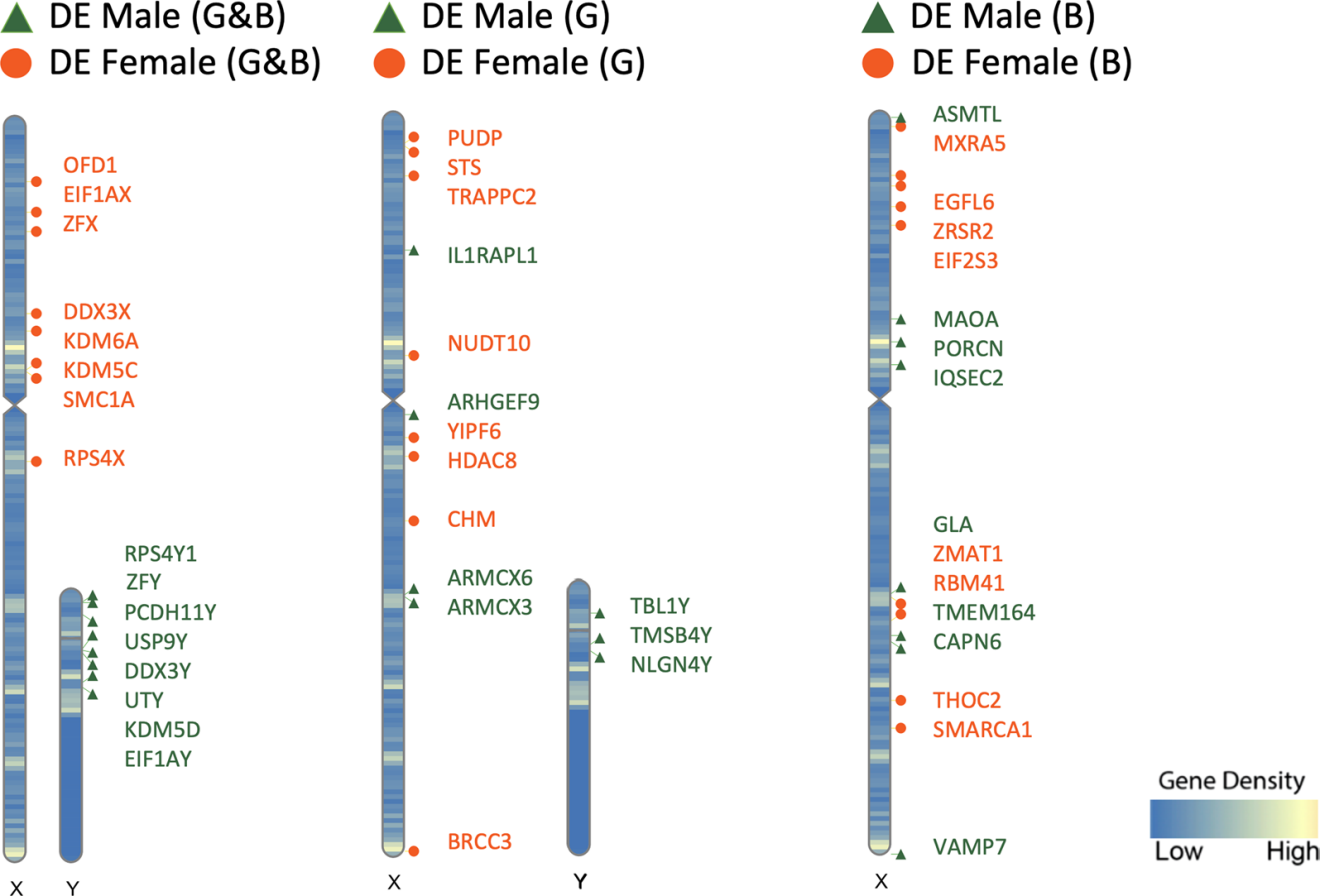
Ideogram visualization of significant (p ≤ 0.05) sex-biased gene expression on sex chromosomes comparing two datasets (G: Gonzalez et al., B: Braun et al.). Includes protein-coding RNA only. M: male (green triangles), F: female (orange circles)

In addition to the potential mutation-masking effect of X mosaicism, XX karyotype comes with another advantage. While the original model of X inactivation held that the inactivated X is functionally silenced outside of the PARs, it is now well established that select transcripts can be expressed from the inactive X, showing biallelic expression in a chromosome dosage-dependent manner [41]. These XCI “escapees” account for a large portion of the genes upregulated in the early XX placenta compared to the XY placenta. In 2018, Gonzalez et al. showed that out of 58 differentially expressed genes in the late first-trimester placenta, over a third were X-linked genes upregulated in female samples, and half of those genes were known to escape X chromosome inactivation [33, 61]. Among this group, *DDX3X, EIF1AX, KDM5C*, *KDM6A*, *OFD1*, *RPS4X, SMC1A*, and *ZFX* were confirmed as upregulated in XX females (Fig. 1) a subsequent analysis suggested that these genes escape X inactivation in chorionic villus (CV) in a robust manner [35]. X-linked genes *BRCC3, CHM*, *EGFL6*, *EIF2S3, HDAC8*, *MXRA5, NUDT10, PUDP*, *RBM41*, *SMARCA1, STS*, *THOC2*, *TRAPPC2*, *YIPF6*, *ZMAT1*, and *ZRSR2* were also found to be upregulated in females in one of the two datasets, indicating that these genes may escape X inactivation in a less robust fashion, potentially varying by cell type. Indeed in a single cell transcriptomic analysis of the maternal–fetal interface, trophoblast cells appeared to have unique X chromosome genes upregulated in females compared to males, which included *MAGEA4* (melanoma associated antigen 4) and *TMSB4X* (thymosin beta 4) [34]. Phung et al. have suggested that while a small group of X genes reproducibly escape inactivation across individuals and tissue regions (*PLCXD1, GTPBP6, PUDP, CSF2RA, SLC25A6, ASMTL, AKAP17A, DHRSX, STS, EIF2S3, ZFX, DDX3X, KDM6A, DIPK2B, UBA1, SMC1A, RENBP, FLN4*), others exhibit variable and heterogeneous escape that varies between individuals and between tissue regions in the same individual (*CD99, EGFL6, RPS6KA3, MBTPS2, SEPT6, CYBB, MED14, USP9X, CDK16, TIMP1, WDR13, MAGED2, OPHN1, EFNB1M, PIN4, RPS4X, ATRX, TSPAN6, ACSL4, PLS3, DOCK11, IL13AR1, Cxorf56, GPC4, HTATSF1, GABRE, BGN, AVPR2, ARHGAP4, HCFC1, IRAK1, MECP2*) [56]. This finding may help explain variability in reports of the specific transcripts that escape X inactivation in the placenta and suggests that escape from X inactivation could act as a tunable protective mechanism, providing unique benefits in different cell types.

It is currently unknown whether XCI escapees play a functional role in the early stages of human placentation, however X inactivation escapees have been shown to contribute to female protection against mitochondrial stressors in human third trimester placenta. Gong et al. [58] demonstrated that the propylamine transferring enzyme spermidine synthase (*SMS*) shows X inactivation escape in term placentas, and that its relative insufficiency in male placenta is associated with vulnerability to mitochondrial stressors. It was observed that polyamine metabolite diacetylspermine is higher in the female placenta and in the serum of women pregnant with a female fetus and correlated both with an increased risk of preeclampsia and a decreased risk of fetal growth restriction (FGR). To our knowledge, this study provides the first direct connection between sex differences in placental gene expression, changes in metabolism, and pregnancy outcome. In addition, Howerton et al. [62] demonstrated that escapee gene O-GlcNAc transferase (*OGT*) mediates female resilience to the effects of maternal psychosocial stress in mice and found its abundance to be higher in human female term placenta as well. OGT and SMS were not found to be significantly differentially expressed in 11- to 16-week CV, however expression patterns indicate a trend toward higher expression in females which is likely to increase over time [35], and may be more pronounced when looking at individual cell types in the CV. Functional studies of early human placentation to identify genes that escape XCI are ethically and technically challenging but will become more feasible given recent advances in 2D trophoblast stem cell models and 3D trophoblast organoid models [63–65].

### The Y chromosome

The Y chromosome is much smaller and gene-sparse compared to the X, with most of its contents primarily involved in spermatogenesis and male fertility [66]. Chief among these is sex determining region Y (SRY), which triggers a developmental cascade that converts the nondifferentiated fetal gonad to testis, leading to the production of androgens such as testosterone from Leydig cells, which then trigger the canonical hormonal masculinization of the male fetus. A subgroup of Y chromosome genes with X homologs outside the pseudoautosomal regions have been shown to be essential for embryonic development, primarily through chromatin modification and RNA splicing [66].

Gene expression from the Y chromosome is detected in early CV samples, with *DDX3Y*, *EIF1AY, KDM5D*, *PCDH11Y*, *RPS4Y1*, *USP9Y*, *UTY*, and *ZFY* expressed consistently in males across datasets (Fig. 1). Additionally, transcripts *NLGN4Y, TBL1Y*, and *TMSB4Y* were detected in a similar analysis which sequenced a broader variety of RNA types [33]. Single cell sequencing of villus tissues collected from late first-trimester healthy pregnancies showed that Y transcripts such as *DDX3Y, EIF1AY, RPS4Y1* were specifically upregulated in male placental cell types including trophoblasts, stromal cells and Hofbauer cells [34].

Importantly, male chorionic villus expresses several Y chromosome transcripts that correspond to peptides that compose the human H-Y antigen, which is detectable in syncytiotrophoblast debris [67]. Several of these Y transcripts correspond to peptides that are presented by the major histocompatibility complex (MHC), including *KDM5D, DDX3Y, ZFY, and UTY*, which were expressed in early CV in two separate analyses (Fig. 1). In single cell analysis, *DDX3Y* was expressed consistently across different cell types such as trophoblasts, stromal cells and Hofbauer cells in early pregnancy with a male fetus [33–35].

### X chromosome expression in the male placenta

While the majority of upregulated genes in early male placenta were Y-linked, three X-linked genes (*ARHGEF9, ARMCX3*, and *ARMCX6*) were also identified as upregulated by Gonzalez et al. [33], perhaps due to upstream Y-linked genes or downregulation of the second X chromosome in females. Interestingly, the protein encoded by *ARMCX3* regulates migration and invasion in tumor cells, functions which are also relevant to placentation. *ARMCX6* was also found to be upregulated in male term placenta [26], suggesting that this trend may persist throughout placental development. Additionally, Braun et al. [35] found the X chromosome genes *CAPN6, GLA*, *IQSEC2*, *MAOA*, *PORCN*, and *TMEM164* to be upregulated in males relative to females. These comparisons suggest that complex sex-linked regulation of gene expression beyond the effect of X dosage compensation likely occurs.

### Autosomal sex differences in early placenta

As expected, the most pronounced differences in gene expression between male and female placentas have consistently been localized to the sex chromosomes, however widespread differences in autosomal gene expression have been detected as well, and account for some of the most dynamic developmental sex differences in gene expression (33, 35) found that 31% of differentially expressed sex chromosome genes detected in the late first trimester were also DE in term placenta tissue. However, there was no overlap in the sex-based DE autosomal genes between early and term placenta, suggesting that sex-selective transcriptional programs correspond to specific developmental stages. This observation highlights the need to examine placental gene expression and function across gestation.

### Chromosomal location of DE genes

Braun et al. [35] found that the group of autosomal genes upregulated in females was distributed widely throughout the genome, while the autosomal genes upregulated in males were clustered in particular loci including Chr11q13.1 (*CYB561A3*, *FAU, GPR137*, *SCYL1*, *TMEM258*, *VPS51*, *YIF1A*), Chr17q25.3 (*C1QTNF1*, *CANT1*, *GAA*, *SLC16A3*, and *TIMP2*), Chr19p13.11–13 (*ATP5D*, *BSG*, *CIRBP*, *CDC34*, *GPR108*, *LSM7*, *MRPL54*, *NDUFA11*), and Chr19q11-13 (*COX6B1, ECH1, EID2*, *LGALS13*, *LGALS14*, *RABAC1*, and *PSG8*). In these latter two loci, the male-upregulated DE genes were enriched for mitochondrial transcripts, as well as placenta and pregnancy specific factors that are particularly important for early placental function. Some of these pregnancy specific factors on chromosome 19q13 have been linked to preeclampsia, including the galectin *LGALS13* (PP13), a putative early pregnancy biomarker for placental dysfunction [68]. Expression from the CGB-LHB cluster at Chr19p13 was also observed to be sex-biased at term [27], but in that case the expression was elevated in females. Localized enrichment of genes upregulated in males was not a prominent feature in the Gonzalez dataset, perhaps due to differences in gestational age (10–13 weeks vs. 11–16 weeks), RNA type (total vs. messenger), analysis pipeline, sample type (CV biopsy vs. elective termination tissue), demographic variability, and/or cell type composition of the samples. Due to these factors, it is important for samples to be as clearly characterized as possible for direct comparison.

### Functional associations of DE genes

**Fig. 2:**
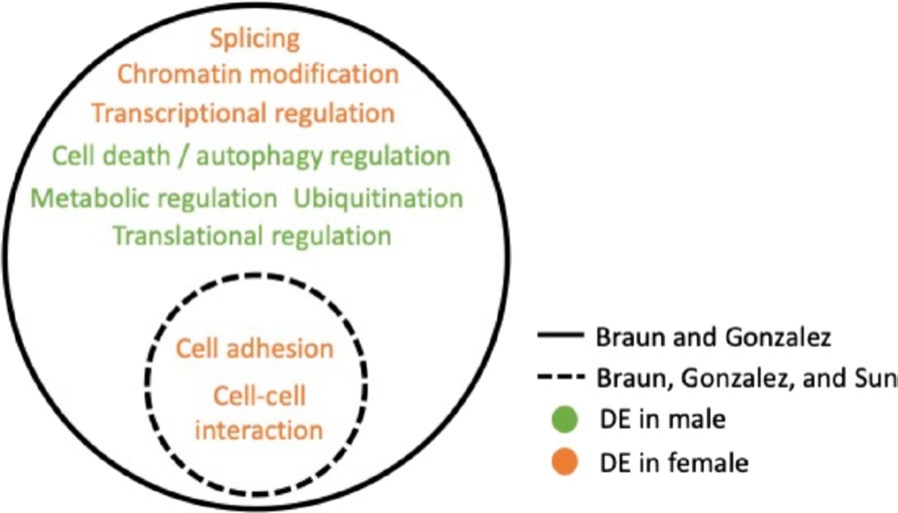
Functional associations of DE transcripts in male CV (green) and female CV (orange)

Multiple analyses of the first-trimester placental transcriptome found chromatin modification, transcription, splicing, translation, signal transduction, metabolic regulation, cell death and autophagy regulation, and ubiquitination were DE between male and female placenta [33, 35]. All current studies of early placenta found sex differences in cell adhesion and cell–cell interaction [33–35], making this functional category one of the most consistent and pronounced sex differences in early human placental transcription (Fig. 2).

In a single cell analysis of paired decidual and placenta samples, 59 differentially expressed receptor–ligand pairs were detected between placenta and decidua in male fetus pregnancies [34]. Male-upregulated placental receptors include *ENG*, *ERBB2* and *INSR*, and upregulated maternal decidual ligands include *COL1A1*, *HSPA5*, and *TGFB1*. Males showed endoglin (*ENG)* upregulation in bulk CV as well [35], a TGF-beta-related integral membrane component that plays an important role in developmental tissue invasion and vascular remodeling [69]. In line with other observations in bulk CV, the male trophoblast transcriptome appeared to be enriched for protein translation, mitochondrial and ribosomal functions [34]. Evidence suggests this sex difference may persist throughout gestation [70, 71], perhaps reflecting enduring differences in energy metabolism, oxidative stress, or hormone synthesis.

### Upstream regulators of DE genes

While transcriptomic datasets may vary at the level of individual genes, robust sex differences in placental gene expression can be best detected at the pathway level [25]. The Ingenuity Pathway Analysis (IPA) Comparison Analysis tool was used to compare Braun et al. [35] and Gonzalez et al. [33] (Table 3). This analysis was restricted to protein-coding RNAs which were included in both datasets and showed appreciable overlap in terms of canonical pathways and upstream regulators. Both datasets highlighted the mTOR pathway as upregulated in males. This matches with observations by Sedlmeier et al. [28, 72] as well as in a meta-analysis of human placenta which found mTOR to be a consistent point of sex differences at term [27]. The mTORC1 and mTORC2 complexes are master regulators of anabolic growth and organism size, and this consistent trend toward male elevation likely contributes to the establishment and maintenance of sexual dimorphism. Males also showed upregulation of the Endocannabinoid Cancer Inhibition related pathway, as well as PPAR, a regulator of energy homeostasis, and NRF2 Oxidative Stress signaling pathways. Females also showed consistency in the predicted upregulation of the ferroptosis cell death pathway, as well as MAP kinase family member ERK5, which has been shown to be critical for vascular development and endothelial function [73, 74]. Predicted upstream regulators that overlap between the datasets included microRNA miR-30c-5p, which exhibits sex-dependent expression in colorectal cancer in relationship to estrogen receptor, as well as mitochondrial enzyme CPT1B, a carnitine palmitoyltransferase that is part of the long-chain fatty acid beta-oxidation pathway, and a MAPK pathway growth regulator PTPRR in females, along with lymphocyte-related immune regulators CD24 in females and CD38 in males.

**Table 3:** Ingenuity pathway analysis (IPA) comparison analysis of Gonzalez 2018 [33] and Braun 2020 [35]

Canonical pathways	Gonzalez 2018	Braun 2020
Category	Z score	
mTOR signaling	1	1.633
Ferroptosis signaling pathway	− 0.816	− 0.816
ERK5 signaling	− 0.447	− 1
Endocannabinoid cancer inhibition pathway	1	0.333
PPAR signaling	1	0.302
NRF2-mediated oxidative stress response	0.447	0.447

Upstream regulators	Gonzalez 2018	Braun 2020
Category	Z score	
ESR1	− 2.927	− 2.822
CD24	− 1.987	− 3.308
CPT1B	− 1.131	− 3.582
miR-30c-5p (and other miRNAs w/seed GUAAACA	1.89	2.796
PTPRR	− 1.987	− 2.28
ASPSCR1-TFE3	2.236	1.897
CD38	1.987	2.123
Mifepristone	1.8	2.142
PKD1	1.48	2.414
AR	− 1.998	− 1.842
GSKJ4	− 1.342	− 2.449
Firre	2	1.706
Tetraethylammonium	− 2	− 1.633
EGF	1.85	1.694
Valproic acid	− 1.37	− 1.977
TRAP1	− 1.342	− 2
Pirinixic acid	0.412	2.899
FN1	− 2.236	− 1.014
TCF4	1	2.1698
XBP1	1.945	1.212
ERBB2	2.709	0.358
SYVN1	2.449	0.577
TSC2	− 1.564	− 1.412
Topotecan	0.853	2.111
BMP4	1.869	1.021
Tamoxifen	1.424	1.373
Benzo(a)pyrene	1.4	1.39
SMARCA5	− 0.378	− 2.236
IGF2BP1	− 0.958	− 1.633
UBQLN2	− 1.387	− 0.816
Mek	− 1.187	− 0.982
MYCL	− 1.98	− 0.104
KLF3	1.155	0.728
Diseases and functions		
Category		
Morbidity or mortality	1.118	3.865
Organismal death	1.039	3.671
Congenital encephalopathy	0.928	2.623
Abdominal cancer	1.557	1.972
Abdominal neoplasm	1.247	2.212
Hematologic cancer	0.872	2.544
Abdominal carcinoma	1.842	1.504
Myeloid or lymphoid neoplasm	0.492	2.764
Neoplasia of blood cells	0.377	2.84
Formation of solid tumor	1.952	1.223
Solid tumor	1.375	1.765
Tumorigenesis of epithelial neoplasm	1.635	1.475
Malignant solid organ tumor	− 1.091	− 1.969
Intraabdominal organ tumor	0.976	2.081
Skin cancer	− 1.067	− 1.98
Development of digestive organ tumor	1.238	1.709
Frequency of tumor	1.687	1.181
Non-melanoma solid tumor	1.217	1.643
Cancer	1.178	1.298
Malignant solid tumor	1.456	0.981
Extracranial solid tumor	1.098	1.309
Growth failure or short stature	0.16	2.221
Incidence of tumor	1.183	1.175
Development of malignant tumor	1.368	0.849
Digestive organ tumor	0.588	1.587
Malignant genitourinary solid tumor	1.719	0.437
Infection by RNA virus	1.067	0.994
Infection of cells	1.319	0.716
Development of carcinoma	1.3	0.623
Infection by HIV-1	1.11	0.729
HIV infection	1.11	0.729
Digestive system cancer	0.368	1.46
Viral infection	0.448	1.367
Head and neck tumor	− 1.308	− 0.437
Skin tumor	− 1.069	− 0.634
Brain lesion	− 0.911	− 0.681
Renal lesion	0.651	0.883
Urinary tract tumor	0.152	1.342
Anogenital cancer	1.193	0.269
Connective or soft tissue tumor	1.331	0.113
Development of urinary tract	− 0.97	− 0.391
Infection of embryonic cell lines	0.137	1.213
Genital tumor	0.446	0.763
Pelvic tumor	0.11	1.04

Activation z‐score represents the inferred activation states of predicted transcriptional regulators based on the IPA molecular network. Positive values = activated in male and/or inhibited in female, negative values = activated in female and/or inhibited in male, (IPA version update 12/13/2020)

Upstream regulators of sexually dimorphic receptor–ligand pairs identified in matched placenta and decidua included cytokines *CSF1*, *IFNG*, *IL1B*, *IL6*, *IL10*, *SPP1*, *TNF*, and *WNT3A* [34]. 32 receptor–ligand pairs were selectively upregulated between female placenta and decidua with the most significantly female-upregulated placental receptor, *ITGB8*, interacting with decidua-expressed ligands *COL4A1*, *FN1*, *LAMA1*, and *LAMB1*. Additionally, *IL36RN* is upregulated in female first-trimester placenta and binds to decidua-expressed *IL1RL2*. This receptor may play a critical role in migration and invasion potential for proper implantation and placentation. This female-specific extracellular matrix-related signature aligns with observations in bulk tissue which found a network of collagens, integrins, and laminins to be selectively upregulated in CV of females [35].

### Sex differences in placental immune genes

Transcriptome profiling of early to mid-gestation placenta reveals that immune signaling is a hub of early prenatal sex differences, a trend noted in all first trimester datasets [33–35]. This observation matches the sex differences in immune signaling in term placenta outlined above. Sex differences in immune-related signaling are pronounced in postnatal life [75], and appear to transcend the unique tissue properties of placenta and the distinct hormonal microenvironment of pregnancy.

In a comparison of 11- to 16-week male and female bulk tissue CV, immunomodulators were identified as some of the most highly differentially expressed [35]. In particular, placenta-specific galectins *LGALS13*, also known as pregnancy associated protein 13 (*PP13*) and *LGALS14*, also known as pregnancy associated protein 13-like (*PPL13*) were upregulated in male CV. Atypical expression of these secreted immunomodulators has been associated with a number of different gestational pathologies including spontaneous abortion and preeclampsia [68, 76] and therefore may contribute to the sex differences noted in these disease processes. LGALS13 and LGALS14 have been shown to induce apoptosis of cytotoxic T cells, and their presence in fetal syncytiotrophoblast suggests that they may act as mediators of placental interaction with maternal immune cells. Some theorize that H-Y antigen recognition by maternal immune cells contributes to elevated rates of secondary recurrent miscarriage following prior male fetus pregnancies [77]. Therefore, it may benefit the survival of a male fetus to establish heightened protection against maternal lymphocyte recognition in the event that H-Y antigens come in contact with maternal lymphocytes and trigger MHC-mediated recognition. It has also recently been demonstrated that LGALS13 polarizes human neutrophils to a growth-promoting regulatory phenotype [78], suggesting that this class of immunomodulators may play a pleiotropic role in tolerizing immune cell types normally associated with rejection and tissue damage.

In the same analysis, *CCRL2* was found to be among the most upregulated in the male placenta. This atypical chemokine receptor subunit increases neutrophil chemoattraction in a mouse model via interactions with CXCR2 [79]. Others have demonstrated an increase in tissue resident neutrophils in the second-trimester decidua, which take on a proangiogenic phenotype and may provide pathogen protection at the fetal–maternal interface [80]. Therefore, it may be useful to consider fetal sex when exploring the role of maternal lymphocytes and neutrophils in pregnancy maintenance and loss, especially since these cells types can be critical determinants of immune tolerance or rejection. It is also interesting to note that in a meta-analysis of transcription in adult neutrophil and T cell-related genes emerged as sex-linked signatures [81], raising the possibility that sex differences observed in early placenta share commonalities with postnatal sex differences in other tissues, despite the unique hormonal and immunologic niche that a developing human placenta inhabits.

A single cell analysis of the interface provided cell-specific insight into sex-dependent immune interactions at the fetal–maternal interface through single cell analyses that examine trophoblasts, stromal fibroblasts, Hofbauer cells, antigen presenting cells, and endothelial cells with single cell RNA sequencing [34]. Sun et al. found that female trophoblasts are enriched in the cytokine-mediated signaling pathway and respond to various compounds and stimuli. Chemokines *CCL3*, *CCL4, CXCL8* were found to be upregulated in female trophoblasts. CCL3 and CCL4 are ligands which bind to decidua-expressed receptors CCR1 and CCR5, involved in recruitment of natural killer (NK) cells and monocyte migration. The authors also found *CCL13* and *RGS1* upregulated in female Hofbauer cells, which may increase M2 phenotype to induce Th2 response. *HLA-C* was upregulated in male trophoblasts, along with *FCGBP*, an IgGFc-binding protein which showed similar male-selective upregulation in bulk tissue [35]. As noted above, an Ingenuity Pathway Comparison Analysis of CV samples highlighted shared upstream regulators, CD24 activated in females and CD38 activated in males, both thought to influence immune cell development and lymphocyte function. Taken together, it appears that chemokine signaling and other mediators of cell–cell immune interaction are a promising area of investigation to understand the nature and impact of sex differences in early human placentation.

### Sex steroid hormones in placenta

Sex differences in early placental development and fetal–maternal interaction can initially be explained by a cell-intrinsic effect of sex karyotype, resulting in differential expression from sex chromosomes and autosomes. Indeed, differences in autosomal transcription in XX and XY conceptuses exist before the onset of testis development [23]. The onset of fetal gonadal hormone production is likely to add a layer of modulation that interacts with cell-intrinsic mechanisms to influence autosomal gene expression in the placenta in complex ways. The role of androgens and their receptors in placenta has recently been reviewed by Meakin et al. [82], with complementary discussion provided here.

**Fig. 3:**
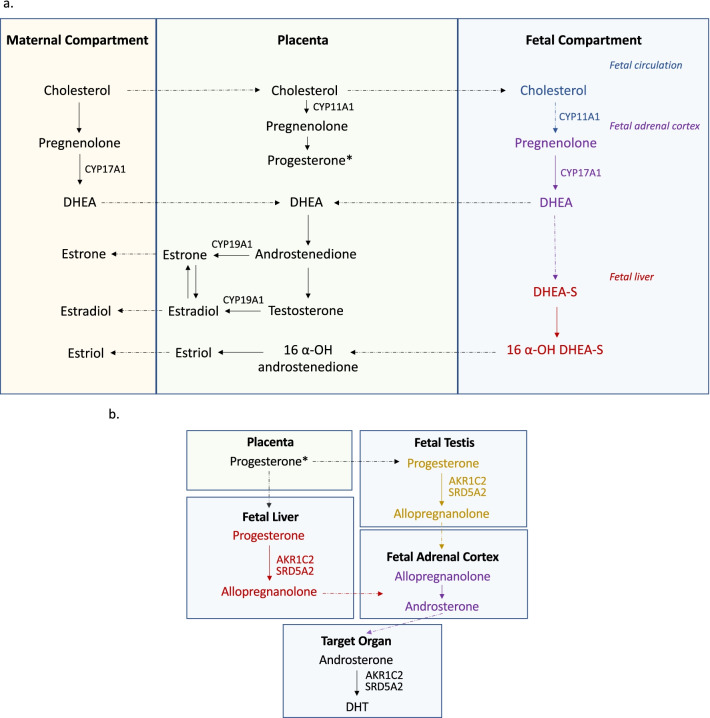
Representation of conventional (**a**) and “backdoor” (**b**) pathways of androgen production. Key enzymes labeled next to corresponding step in pathway. Dotted arrows indicate transport of steroid hormones and intermediates between compartments/organs. DHEA, dehydroepiandrosterone; DHT, 5α-dihydrotestosterone

Regulation of the gestational hormonal environment operates as an axis split between the maternal, placental, and fetal compartments (Fig. 3a). Rate-limiting enzymes and their substrates are segregated between these compartments to allow precise control of biosynthesis, which depends on the maternal hypothalamic pituitary axes, the syncytium of the chorionic villus, as well as the fetal liver, adrenals, and gonads. [83, 84]. Sulfonation of steroids in the fetal compartment is a major mechanism of sex steroid control that renders both estrogens and androgens largely inactive in fetal circulation by inhibiting the steroid hormones from crossing the cell membrane to influence transcription. Steroid sulfatase (STS) is highly expressed in the placenta and catalyzes the conversion of sulfated steroid precursors to the unconjugated active form [85]. STS is located on the X chromosome and appears to escape inactivation, showing elevated expression in the first-trimester female placenta [33]. STS provides a direct link between sex karyotype and sex-dependent hormone signaling in placental tissue and is a promising candidate for future investigation of placental sex differences.

In both studies of the first trimester CV transcriptome, Cytochrome P450 subfamily 11 alpha 1 (CYP11A1) is significantly upregulated in male placenta [33, 35]. This syncytiotrophoblast-specific enzyme performs the rate-limiting step in the conversion of maternally derived cholesterol to pregnenolone, which acts as the precursor to all steroid hormones including estrogens and androgens. It is possible that this upregulation in males is related to the role pregnenolone plays as a precursor to testosterone in the fetal testes, however this is only one of many possibilities that should be examined further.

During the first trimester of male prenatal development, placental human chorionic gonadotropin (hCG) induces the differentiation of testicular mesenchymal cells into Leydig cells and stimulates testosterone production, the control of which is taken over by the fetal pituitary gland in the ninth gestational week [86]. Circulating testosterone levels in fetal plasma are correlated with biosynthesis in the fetal testis beginning around gestational weeks 8–10, suggesting that this is the primary canonical source for fetal androgens at this stage [87]. Secretion of testosterone from the fetal testes into fetal circulation begins around week 10 and peaks in the middle of the second trimester at week 16, reaching concentrations similar to a post-pubertal male. Endocrine crosstalk between fetal testes and the placenta is an essential part of sexual differentiation [88], however it has not yet been established whether circulating fetal testosterone has a reciprocal influence on placental function during the mid-gestational androgen peak.

An alternative androgen production pathway involving the placenta has been proposed by O’Shaughnessy et al. [89]. In this “backdoor” pathway, placental progesterone gives rise to androsterone that replaces testosterone as the source of circulating androgen precursor, which is converted by AKR1C2 and 5 alpha reductase (SRD5A2) to dihydrotestosterone (DHT) in the target tissue (Fig. 3b). Sex differences were observed in concentrations of fetal testosterone and fetal androsterone in second trimester fetal blood from electively terminated pregnancies. The placental compartment is likely required but not sufficient in the androsterone to DHT conversion process, which also depends on enzymatic conversions in fetal liver, adrenals, and testes in a multi-step process. Placental syncytiotrophoblast does express the androgen receptor (AR) that binds to DHT, and AR is expressed at low but similar levels between sexes in 11- to 16-week placentas [35]. The enzymes SRD5A2 and AKR1C2 are necessary for the local conversion of circulating precursors to active DHT via the canonical and non-canonical pathways, respectively, and their expression was undetectable in CV, suggesting that the developing CV is not able to effectively convert precursor hormones to DHT locally. Placental aromatase Cytochrome P450 Family 19 Subfamily A Member 1 (CYP19A1) is expressed highly in both sexes with no difference between fetal sexes from 11 to 16 weeks, however the abundance of the protein products of these genes was not quantified. It is therefore possible that fetal DHT is being produced locally and signaling through AR occurs at a low level in the placenta at this stage., This capacity may increase over time and come under the influence of fetal sex through yet uncharacterized mechanisms [90].

Maternal androgens also increase over the course of gestation at levels that are similar between male and female-fetus pregnancies, but are elevated in male preeclamptic pregnancies compared to normotensive pregnancies of both sexes and female pregnancies with preeclampsia [91]. Maternal androgens are normally buffered from fetal circulation via conversion to estrogen by CYP19A1 to prevent inappropriate virilization [90]. It is probable that the DHT bound by placental AR is maternal in origin, as uterine tissues have been shown to express SRD5A2 more highly than placenta itself [92], and the AR is localized specifically to the syncytium, which is in direct contact with maternal blood.

While the means by which fetal sex influences this type of hormone receptor signaling remains to be established, observations by Sun et al. support a role for fetal androgens driving sex differences in gene expression at the maternal/fetal interface via the upstream regulator DHT binding to AR and impacting sexually dimorphic genes in the trophoblast population. Additionally, a comparison analysis of Gonzalez and Braun with the updated IPA software limited to protein-coding RNA does show both AR and Estrogen Receptor (ESR) among the list of upstream regulators (Table 2). Perhaps contrary to expectations, both AR and ESR signaling was shown to be activated in females and/or inhibited in males in both datasets. By the end of the first trimester, the placenta takes over from the corpus luteum as the primary source of the estrogens estradiol (E_2_) and estriol (E_3_). While sex differences in the abundance and influence estrogens have not yet been reported in the developing placenta, this common finding suggests that a more direct investigation of the placental compartment may uncover some, perhaps related to previously outlined sex differences in gene expression of regulatory genes like STS and CYP11A1 [33, 35].

Much of what is known about fetal hormone levels is derived from measurements in cord blood. Interestingly, when hormone levels in term cord blood were compared with levels in the placenta by Sedlmeier et al. [28], they found that while levels of free testosterone in cord blood were significantly higher in males than females as expected, these levels did not correlate with those in placental tissue itself. In the placenta, testosterone levels were actually observed to be significantly higher in females than males in pregnancies exposed to an n-3 long chain polyunsaturated fatty acid (n3LCPUFA) enriched diet. Authors also observed an elevated estradiol/testosterone ratio in males from the same treatments, which they concluded to be driven by elevated testosterone in females and not by differences in estradiol. These findings run contrary to predicted associations between karyotypic sex and sex steroid hormone levels and suggest that hormone measurements in cord blood may not be the best proxy for levels in placenta itself.

Of the genes Sedlmeier et al. [28] found to be differentially expressed by sex in term placenta, only one gene in the Wnt family correlated moderately with estradiol levels. None of the DE genes contained androgen response elements or correlated with testosterone levels, while four genes in the Wnt family contained estrogen receptor alpha response elements. These data do not support a strong role for sex steroid driven expression in placenta at term, however this stage of development is not characterized by pronounced sex differences in hormone levels and it is possible that hormone-driven expression is more detectable at earlier stages, as indicated by the proposed upstream regulators in Table 2. Observations from week 11 to 16 suggest that sex differences in autosomal gene expression are unlikely to be explained solely by the impact of gonadal fetal androgen exposure, as sex differences over this gestational window do not correlate strongly with the predicted course of the fetal testosterone peak in males [35]. However, given that androgen production can vary in timing and plasma concentrations between individuals [88, 93], circulating levels may not represent the levels in CV tissue, and hormone-driven responses may vary by cell type, therefore potential correlations may be difficult to detect. Further examination of sex differences in steroid hormone dynamics in the placenta will be necessary to solve this puzzle, with particular attention paid to the role of hormone synthesis and sulfonation in the placenta itself.

### Sex differences in microRNA in human placenta

Consisting of single-stranded non-coding RNA molecules approximately 22 nucleotides in length [94], microRNAs (miRNAs) regulate mRNA transcription and translation [95]. MiRNAs are enriched on the X chromosome and have been shown to be responsive to estrogens [96], suggesting that sex differences in miRNA expression can be produced both by the previously outlined mechanisms of XCI escape, as well as by sex hormone mediated transcription. MiRNA have emerged as new frontier in the study of placenta sex, with two recent studies on term placenta reporting differences in miRNA expression in male and female term placenta. Guo et al. [97] found 32 miRNA differentially expressed, with the male-selective transcripts annotated as evolutionarily younger and enriched in endocrine functions, and female selective transcripts enriched for the imprinted miR-379 cluster on Chr14 (C14MC) which linked to estradiol, glucocorticoids, and brain-specific mRNA targets. In their study of the interaction of placental sex and maternal n-3 LCPUFA dietary supplementation, Sedlmeier et al. [72] highlighted miR-99a as differentially expressed by sex and modulated by maternal diet, linking its expression to sex differences in mTOR-related mRNA expression. The link to mTOR may help to explain the mechanisms underlying sexual dimorphism in fetal growth. A sex-linked upstream regulator of miR-99a has yet to be identified, and it remains possible that these differences could be downstream of hormone signaling or sex karyotype.

In the largest miRNA sequencing study to date of healthy first-trimester and third-trimester placentas, Flowers et al. found 11 and 4 miRNAs differentially expressed by sex in each trimester, respectively, all elevated in female-fetus pregnancies. Six of these miRNAs [4 first trimester, 2 third trimester] had loci on the X chromosome, but the majority were on autosomes. Greater sexual dimorphism was present in the first trimester and one X-linked miRNA, miR-361-5p, was significant in both the first and third trimester [36]. While the sexually dimorphic microRNAs that have been detected in placenta are mostly located in autosomal loci, the highest density (10%) of human microRNAs are located on the X chromosome. It is possible that an X dosage effect may contribute to observed sex differences as well [98]. Comparative analysis of first versus third-trimester placenta in female only and male only cohorts found 52 female exclusive and 32 male exclusive miRNAs differentially expressed across gestation, including miRNAs from the placenta-specific clusters, C14MC and C19MC [36]. These clusters are large, imprinted miRNAs whose members are principally expressed in placenta, and are maternally and paternally expressed, respectively [99]. In the first trimester, miRNA expression from C14MC was more represented in males and C19MC was more represented in females. This trend was reversed in the third trimester [36]. Overall in first and third trimester, the current collection of observations suggest that microRNAs are a consistent hub of sex differences throughout gestation and are linked to cell signaling, growth, cancer, and immune function pathways.

### Placental sex, biomarkers, and prenatal testing

Understanding how sex manifests early in placental development has the potential to refine prenatal testing and improve the reliability of biomarker screening. Multiple groups have observed higher levels of hCG in maternal blood in the presence of female compared to male fetuses at different stages of pregnancy [[100–105], but see [106] in the case of IVF]. In the second trimester, inhibin-A was shown to be elevated in karyotypically normal female fetuses, which has the potential to reduce the accuracy of inhibin-A based Down syndrome screening in females [107]. In a related observation, Larsen et al. [100] found elevated PAPP-A in serum from female-fetus pregnancies relative to male fetuses from 11 to 14 weeks, noting that this difference could also potentially confound screening for Down Syndrome in females. This is corroborated by observations that the trajectory of PAPP-A, PAPP-A2, INHBA expression in human CV showed sex-dependent trends in expression from 11 to 16 weeks [35]. Brown et al. [16] have demonstrated sex differences in first-trimester placental biomarkers including soluble fms-like tyrosine kinase 1 (s-Flt1), placental growth factor (PLGF) and plasminogen activator inhibitor-2 (PAI-2) in both normal and pathological pregnancies. PLGF, PAI-2, and s-Flt1 were all noted to be higher in the first-trimester plasma in pregnancies with female fetuses, however these sex-specific differences disappeared in the presence of vascular complications such as PE and FGR. In pregnancies affected by hyperhomocysteinemia, plasma levels of placentally derived PLGF and PAI-2 decreased in the case of male fetuses, an effect not seen in female fetuses [16]. This example illustrates one of the ways that sex differences can manifest in pregnancy: the disappearance of a normally occurring dimorphism in pathological states [108]. Once the landscape of human prenatal sex differences and dimorphism is fully mapped in normal pregnancy, the absence of an expected sex difference may itself act as an indicator of future pathology.

With the advent of non-invasive prenatal testing (NIPT) using cell-free DNA, it is now possible to determine fetal sex as early as 7 weeks of pregnancy [109]. The development of screening frameworks that are sensitive to fetal sex will likely improve the predictive utility of both current and future diagnostic tools, as grouping sexes together when sex-biased distributions exist adds noise and masks potentially informative correlations [108]. As NIPT improves, quantitation of placental cell-free RNA transcripts from maternal blood may one day become a standard part of prenatal screening [110], in which case an accurate map of sex differences in the first and second trimester placental transcriptomes will become invaluable in charting sex-specific trajectories over the course of pregnancy to identify the most reliable biomarkers of pregnancy outcome.

### Evolutionary perspective: why consider sex in early human placental development?

**Fig. 4:**
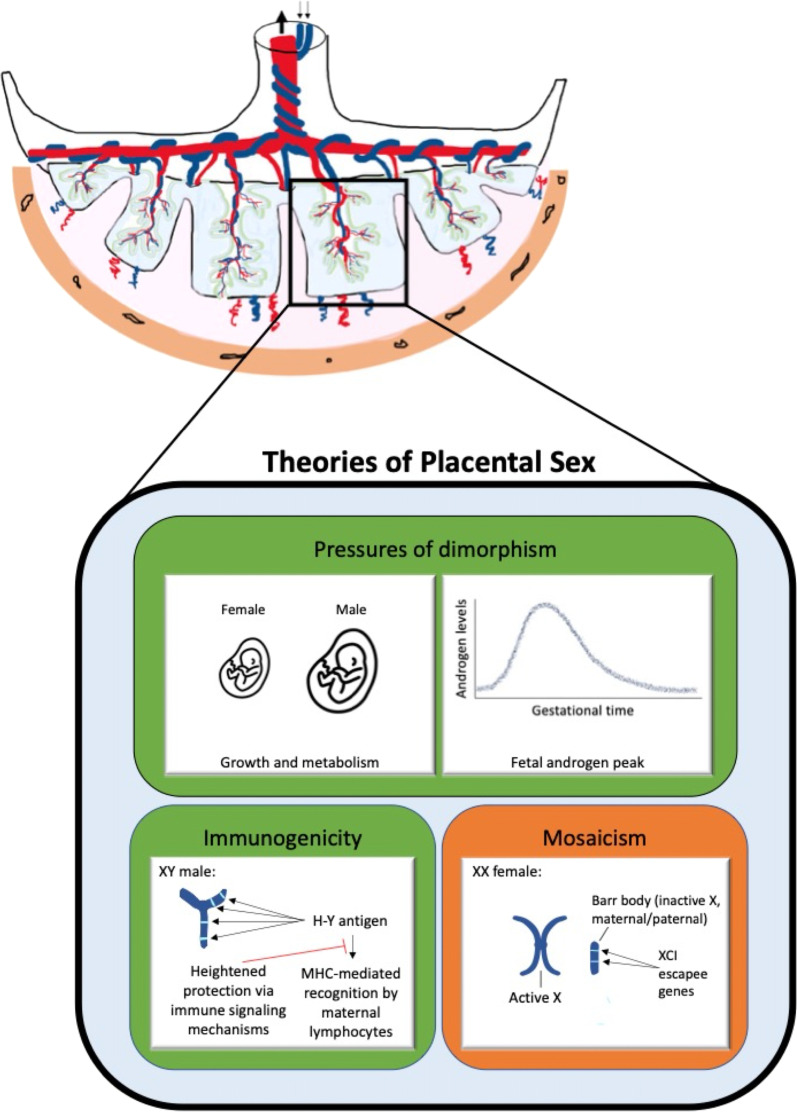
Summary: theorized factors contributing to sex differences in placental gene expression and functions. Green = unique to XY males, orange = unique to XX females

Why, from an evolutionary or developmental perspective, should male and female extraembryonic tissues differ at all? Ultimately, we cannot know precisely which historical evolutionary forces shaped the sex differences we observe in fetal and placental development, but we can encapsulate the current theories that help to generate experimental predictions (Fig. 4).

- The pressures of dimorphism: In a sexually dimorphic species like *Homo sapien*s, males become larger than females on average. The Trivers–Willard hypothesis of differential parental investment in male and female offspring proposes that polygynous mammalian species exhibiting sexual dimorphism can adjust offspring sex ratios adaptively, prioritizing male offspring in high-nutrient environments and female offspring in low nutrient environments [111]. In this framework, males are a high-risk/high-reward investment, producing many F2 offspring when successful in competing for mates, and producing none if unsuccessful, making body size an essential developmental priority. Females of these species tend toward a lower but consistent lifetime parity regardless of size, making them a safer investment for maximizing F2 offspring in adverse environments. This theory was applied to prenatal and placental biology by Eriksson and Clifton, respectively, in 2010 [112, 113] and has provided a guiding heuristic for conceptualizing placental sex differences and their impact on fetal outcomes. In this model, fetal body growth is prioritized by male placenta, while adaptability and survival is favored by females. Observations of sex differences in mTOR-related signaling, mitochondrial function, and energy homeostasis may arise as a result of this selection pressure.
- The foreign Y: XY conceptuses produce foreign alloantigens from the Y chromosome to which maternal XX immune cells are naïve. For this reason, the male fetus may have evolved to interact with the maternal immune system in a way that prioritizes protection from recognition. Given the emergence of immune signaling as a hub of sex differences in the early placental transcriptome, it is possible that the selection pressures shaping lifelong sex differences in immune function may arise in part from the necessities of prenatal survival, particularly at the site of immunological interaction in the extraembryonic compartment. The most consistent of these differences are likely to be driven by chromosomal mechanisms, while those that vary with age are likely to interact with hormonal sex as well [75]. The observed sex differences in cytokine, chemokine, and immunomodulator expression may have arisen in response to this selection pressure.
- XX flexibility: Current evidence suggests that X inactivation is not fully paternally imprinted in human placenta, instead exhibiting a form of large-scale clonal mosaicism with a slight bias towards maternal X expression (54, 56). In addition to random and/or clonally skewed XX mosaicism acting as a buffer against deleterious mutations, X inactivation escape allows for elevated dosage of specific X transcripts in a cell and tissue-specific manner. The loosening of parent-specific imprinting and X gene-dosage limitations in extraembryonic tissues has allowed for the rise of XX protection mechanisms that have been shown to contribute to female resilience against certain metabolic stressors [58, 62]. Observations of consistent and cell-specific groups of X inactivation escapees in placental cells may have arisen as a result of this selection pressure.

### Perspectives and significance

The successful establishment of the fetal–maternal interface is the foundation for a healthy pregnancy. Differences in placental development and function driven by biological sex are likely to contribute significantly to sex differences in adverse pregnancy outcomes in the short term, as well as sex-biased disorders of development in the long term. Understanding what these differences are, how they change throughout gestation and what mechanisms account for them will provide key insight into molecular targets that can be modulated to improve sex-biased obstetrical complications.

It is essential to map the landscapes of sex differences in both health and disease, as a full understanding of the normal state will assist in determining variations associated with disease that may be sexually dimorphic. While a sex difference that emerges in a disease state can indicate that sex-linked mechanisms are at play, it is likely just as important to pay attention to sex differences that exist at baseline in healthy pregnancies and disappear in disease states. If male and female fetuses employ unique transcriptional and translational adaptations under normal conditions, the convergence of the sexes and the absence of difference can, in itself, be an indication that sex-biased pathological outcomes may result [108]. This will be especially important when developing biomarkers for pregnancy monitoring, as well as defining male-specific metabolic and immunologic adaptations that may protect and facilitate their growth and greater relative allogeneicity to their female host.

Discussion of biological sex tends to focus on sex differences that exist in the somatic tissues of postnatal boys, girls, men, and women. With growing recognition of the fetal origins of health and disease, it is important to shed light on sex differences in early prenatal development, as these observations may unlock insight into the foundations of sex-biased pathologies that emerge later in life. More research is needed to determine the impact of both sex karyotypes and gonadal hormones on cell and tissue function in human placenta. In order to understand how fetal sex shapes disease, we must thoroughly map the landscape of sex differences in normal prenatal development.

## Data Availability

Not applicable.

## Abbreviations

PE: Preeclampsia
PPROM: Preterm premature rupture of membranes
IUGR: Intrauterine growth restriction
SGA: Small for gestational age
DE: Differentially expressed
PAR: Pseudoautosomal region
XCI: X chromosome inactivation
CV: Chorionic villus
FGR: Fetal growth restriction
IPA: Ingenuity pathway analysis
MHC: Major histocompatibility complex
NK: Natural killer
hCG: Human chorionic gonadotropin
DHT: Dihydrotestosterone
AR: Androgen receptor
ESR: Estrogen receptor
n3LCPUFA: N-3 long chain polyunsaturated fatty acid
miRNA: MicroRNAs
NIPT: Non-invasive prenatal testing

## Glossary

Chorionic villus: Tree-shaped outgrowth of fetal placental tissue that contacts maternal blood and facilitates exchange across the interface.
Conceptus: All tissues arising from a fertilized egg, including embryonic and extraembryonic compartments.
Decidua: The modified endometrial lining in, which forms the maternal part of the placenta. Decidua is formed in a process called decidualization under the influence of progesterone.
Dosage compensation: A process by which gene expression is equalized between cells of different sexes, involving X Inactivation in mammals.
Eutherian: A subclass of mammals that form a placenta and reach an advanced state of development before birth.
Monotreme: Subclass of mammals that lay eggs, including platypus and echidna.
Parturition: The process of childbirth including dilation, expulsion of the fetus, and separation of the placenta.
Pseudoautosomal region: Paired sequences of nucleotides on the ends of the X and Y chromosomes that are expressed in a dose-dependent manner and inherited like autosomal genes.
Syncytiotrophoblast: Cytotrophoblast cells that have fused to form the multinucleated epithelial outer lining of the chorionic villi.
Tolerance: Prevention of an immune response against a particular antigen.
X inactivation escapees: Genes that are expressed at a detectable level from the otherwise inactivated X chromosome.
